# Case report: Elevation variability and pacing regulation in an elite half-marathon runner: a longitudinal case study

**DOI:** 10.3389/fphys.2026.1793257

**Published:** 2026-04-02

**Authors:** Gerasimos V. Grivas, Walaa Jumah Alkasasbeh

**Affiliations:** 1 Physical Education and Sports, Division of Humanities and Political Sciences, Hellenic Naval Academy, Athens, Greece; 2 Department of Physical Education, School of Sports Sciences, The University of Jordan, Amman, Jordan

**Keywords:** elevation profile, elite athlete, endurance performance, half-marathon, pacing strategy

## Abstract

Pacing strategy is a central determinant of endurance running performance. While elevation profile is known to influence pacing in trail and mountain running, its potential role in road races characterized by moderate elevation variability remains insufficiently examined. This longitudinal case report aimed to explore how differences in course elevation characteristics were associated with pacing patterns and performance outcomes in an elite half-marathon runner. Ten official half-marathon performances completed over a four-year period (2014–2017) by a single elite male athlete were retrospectively analyzed. Elevation-related metrics (total ascent, total descent, and elevation range) and split times (0–10 km and 11–21 km) were examined descriptively to characterize pacing patterns, classified as positive or negative split. Five races were classified as positive split and five as negative split. Finishing times ranged from 69.18 to 87.05 min, and elevation range varied between 26.3 and 116.8 m. The fastest performance occurred during a negative split on a near-flat course; however, pacing classification and elevation characteristics were not uniformly aligned with performance outcomes across races. Within comparable elevation ranges, both positive and negative pacing profiles were observed, accompanied by substantial variation in finishing times. These findings indicate that moderate elevation variability may interact with pacing regulation in a context-dependent manner rather than acting as an isolated determinant of performance. The results highlight the multifactorial nature of pacing behavior in elite half-marathon competition and underscore the value of individualized longitudinal analyses for understanding performance execution in real-world racing conditions.

## 1 Introduction

Pacing strategy represents a fundamental component of endurance running performance, particularly in events lasting between 60 and 90 min, such as the half-marathon. It reflects the athlete’s regulation of effort across race segments with the aim of optimizing speed while managing fatigue (Foster et al., 1994; Abbiss and Laursen, 2008). Distinct pacing profiles—including positive pacing (gradual deceleration), negative pacing (progressive acceleration), and even pacing (uniform effort)—have been described across endurance events, with observational evidence indicating that negative or even pacing is more commonly associated with superior performance outcomes, especially among elite runners (Hanley, 2015; Hettinga et al., 2017). In elite half-marathon competition specifically, pacing has also been described as U-shaped, characterized by a relatively fast opening phase, mid-race stabilization, and a final end spurt (Casado et al., 2021). Importantly, this description is derived from analyses of elite race performances, suggesting that pacing regulation at this competitive level may differ from patterns typically observed in recreational cohorts.

Pacing behavior is shaped by a complex interaction of internal and external factors, including physiological capacity, psychological regulation, environmental conditions, and course characteristics. Among these, course elevation profile represents a practical variable that may meaningfully influence pacing decisions, yet has received limited attention in the context of road racing. In the present context, elevation-related factors of interest include total ascent and total descent, reflecting cumulative mechanical and metabolic load, as well as elevation range (maximum–minimum elevation), which captures variability in terrain profile. Even modest vertical displacement can increase the energetic cost of running and alter stride mechanics, neuromuscular recruitment patterns, and cardiorespiratory strain (Minetti et al., 2002; Snyder et al., 2012; Vernillo et al., 2017), thereby challenging an athlete’s ability to sustain an intended effort distribution. Contemporary integrative models conceptualize pacing as a dynamic regulation process emerging from the interaction of physiological capacity, fatigue resistance, and perceptual control (Tucker and Noakes, 2009; Mauger, 2014). However, how specific elevation-related characteristics within moderately undulating road races interact with this regulatory process at the individual elite level remains insufficiently understood (Grivas et al., 2026).

In trail and mountain running, uphill and downhill sections are known to elicit acute biomechanical and physiological responses that influence pacing behavior (Hoffman and Wegelin, 2009; Jaén-Carrillo et al., 2025). In particular, downhill running is associated with increased eccentric muscle loading, which may contribute to exercise-induced muscle damage, transient reductions in neuromuscular function, and increased muscle soreness—factors that can impair subsequent performance (Bontemps et al., 2020). Although these mechanisms are well established in off-road disciplines, their relevance to urban road races, often classified as “flat” despite featuring gentle undulations, remains insufficiently explored.

Emerging observational evidence suggests that runners who maintain consistent or negative pacing on courses with mild elevation variability tend to achieve superior performance compared with those exhibiting greater pacing fluctuation (Ristanović et al., 2023). In longer endurance events such as marathons and ultramarathons, course elevation has been identified as an important determinant of finishing time, in some cases exceeding the predictive value of demographic or training-related variables (Tucker and Noakes, 2009). While the physiological demands of the half-marathon differ in magnitude from those of the marathon and ultramarathon, several regulatory mechanisms, such as terrain-induced alterations in energetic cost and neuromuscular load (Minetti et al., 2002; Snyder et al., 2012; Vernillo et al., 2017), as well as perceptual feedback processes involved in pacing regulation (Tucker and Noakes, 2009; Mauger, 2014), are likely to operate across endurance durations. Accordingly, even modest elevation variability may meaningfully influence effort distribution and late-race fatigue development in elite half-marathon competition (Hanley, 2016; Casado et al., 2021). Nevertheless, studies specifically examining how moderate elevation variability may relate to pacing patterns in elite half-marathon runners are scarce.

Much of the existing literature is based on cross-sectional analyses or large cohorts of recreational runners (Abbiss and Laursen, 2008; Hettinga et al., 2017). Conceptual frameworks have highlighted that pacing regulation is highly individualized and context-dependent, suggesting that longitudinal, single-athlete analyses may offer unique insight into real-world pacing behavior that cannot be captured through group-level designs alone (Hettinga et al., 2017; Grivas et al., 2026). In this regard, longitudinal case studies, such as Tjelta’s analysis of the 2012 European 1500 m champion, have demonstrated the value of within-athlete approaches for understanding elite performance adaptation under real-world competitive conditions (Tjelta, 2013).

In half-marathon research, performance is commonly examined using first- versus second-half comparisons to evaluate global effort distribution and fatigue development across the race (Hanley, 2016; Casado et al., 2021). This approach provides an ecologically valid framework for distinguishing positive and negative split patterns, particularly in longitudinal single-athlete designs focused on overall pacing regulation rather than kilometer-by-kilometer variability.

Accordingly, the purpose of the present longitudinal case report was to describe how moderate differences in course elevation profile were associated with pacing patterns and performance outcomes in a single elite half-marathon runner. Data from ten certified road races completed over a four-year period were examined, with particular attention to pacing behavior across the first (1–10 km) and second (11–21 km) race segments.

## 2 Materials and methods

### 2.1 Participant

This longitudinal case report analyzed ten official half-marathon performances completed by a single elite male distance runner over a four-year period (2014–2017), during which the athlete was aged 29–32 years. During this period, the athlete had a body mass of 63.4 kg, a height of 1.78 m, and a body fat percentage of 5.4%, assessed using dual-energy X-ray absorptiometry (DXA). Personal best performances included 32:21 for 10 km, 1:09:18 for the half-marathon, and 2:31:20 for the marathon. Laboratory testing conducted within the same competitive period indicated a maximal oxygen uptake (VO_2max_) of approximately 71 ml·kg^−1^·min^−1^ and a lactate threshold running speed of approximately 3:30 min·km^−1^ (~17.1 km·h^−1^). These laboratory measurements were obtained once at baseline to characterize the athlete’s physiological profile and were not reassessed during the subsequent races, as the focus of the study was on pacing behavior across competitive contexts rather than longitudinal physiological adaptation.

### 2.2 Race data and pacing classification

Race data were retrospectively collected from ten certified half-marathon road races completed by the athlete. All races were conducted on asphalt surfaces within urban environments and were characterized by moderate elevation variability. To facilitate interpretation of pacing responses across races, moderate elevation variability was operationally defined as total elevation changes exceeding those typically observed in flat urban half-marathon courses (generally < 20–30 m total variation) while remaining substantially below profiles characteristic of mountainous or trail races (> 150–200 m total elevation change), consistent with descriptions in road-running and terrain-related performance literature (Hanley, 2016; Vernillo et al., 2017; Casado et al., 2021). For each race, the following variables were extracted: total ascent and descent (m), minimum and maximum elevation, elevation range (maximum–minimum elevation), average pace (min·km^−1^), mean running speed (km·h^−1^), and split times for the first (1–10 km) and second (11–21 km) race segments. Finish time was recorded in minutes. Pacing strategy was classified descriptively as either positive split or negative split. A race was classified as a positive split when the second segment (11–21 km) was completed at a slower pace than the first (1–10 km), whereas a negative split was defined as a faster second segment relative to the first. Split times were obtained from GPS-derived data for all races (n = 10). Previous studies have demonstrated acceptable agreement between GPS-derived pacing metrics and official race timing in road-running conditions, with small measurement error unlikely to meaningfully affect split-based pacing classification. Therefore, any potential influence on measurement consistency in the present descriptive analysis is considered minimal. Elevation variability across races primarily reflected mild urban topographical features, such as gradual inclines, overpasses, and short rolling sections, rather than substantial altitude changes or mountainous terrain. All courses were officially certified for road racing and did not include trail or off-road segments.

### 2.3 Data analysis

Data were analyzed descriptively to explore patterns of pacing behavior and performance across races with differing elevation characteristics conducted between 2014-2017. Given the single-athlete longitudinal design, inferential statistical testing was not performed due to the absence of statistical power for between-subject inference. Instead, a descriptive analytical approach was intentionally adopted to examine within-athlete pacing variability across races with differing elevation profiles, consistent with exploratory single-case performance analyses in elite endurance research. All races were completed during competitive phases of the season under typical temperate road-race conditions, thereby limiting extreme environmental confounding. Elevation-related variables included total ascent, total descent, and elevation range (maximum–minimum elevation), allowing evaluation of both cumulative mechanical load and terrain variability. Results are presented as means and ranges to illustrate within-athlete variability across pacing strategies. Comparisons between positive- and negative-split races were used to highlight observed trends rather than to test statistical hypotheses. Associations between elevation characteristics and pacing behavior were examined qualitatively at the individual level to support interpretation of longitudinal patterns within the same athlete. Given the limited number of races analyzed (n = 10), elevation characteristics were examined as continuous variables rather than being categorized into small, moderate, or large classes, in order to avoid imposing arbitrary thresholds. Similarly, pacing classification was retained as a directional descriptor (positive vs. negative split), while proportional segment differences were reported to illustrate that several performances approached near-even pacing. This approach was adopted to preserve descriptive transparency without overstating categorical distinctions. All analyses and visualizations were performed using Jamovi software (version 2.3.28.0).

## 3 Results

**Figure 1:**
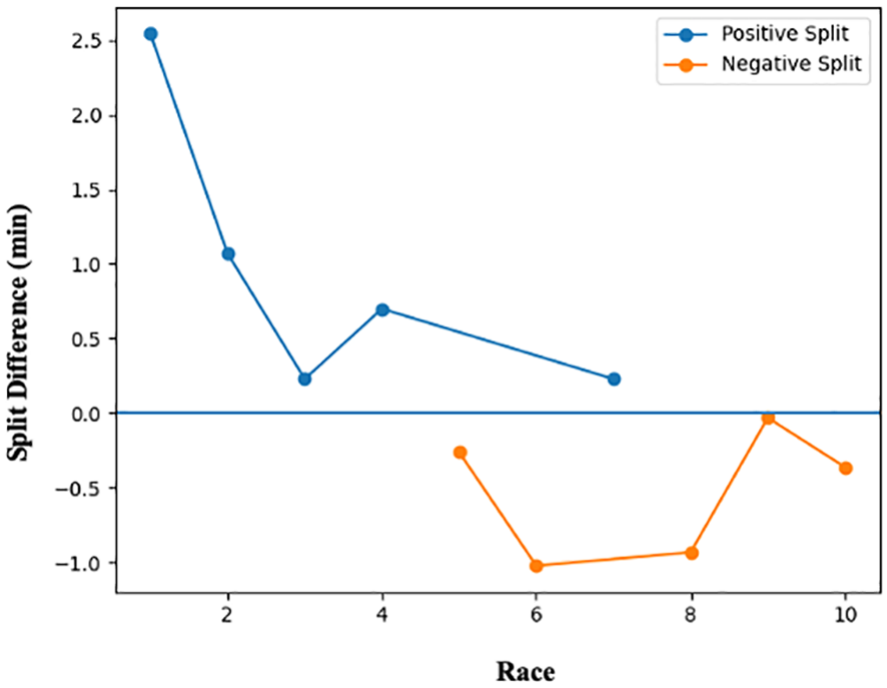
Split difference across ten half-marathon races by pacing strategy. Split difference (11–21 km minus 0–10 km) is shown for each race. Values above zero indicate a slower second half (positive split), whereas values below zero indicate a faster second half (negative split). Races are presented sequentially to illustrate within-athlete pacing variability across different competitive contexts.

**Figure 2:**
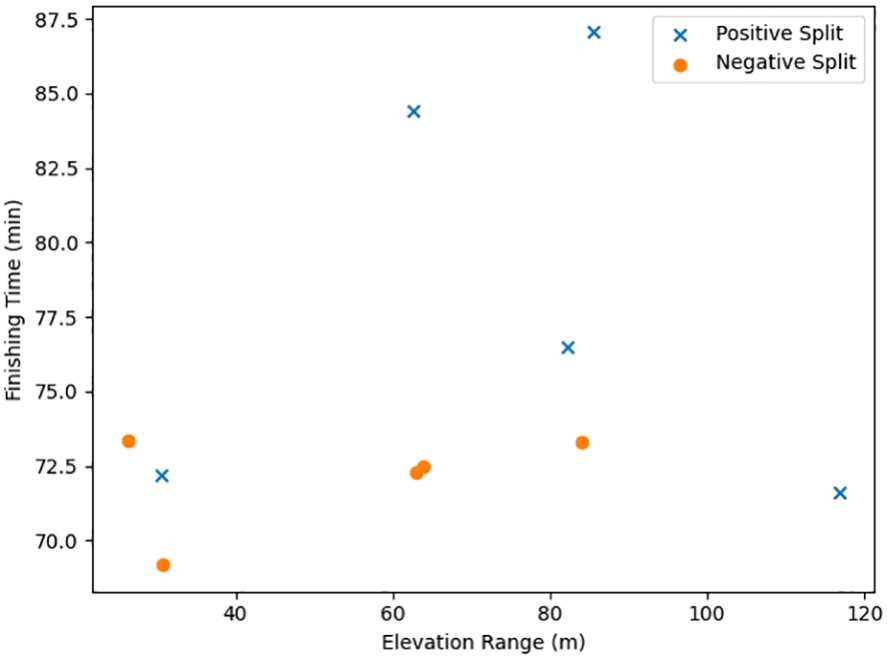
Relationship between elevation range and finishing time across ten half-marathon races. Each point represents one race completed by a single elite runner. Blue markers indicate races performed with a negative split, whereas red markers indicate races performed with a positive split.

Ten official half-marathon performances completed by a single elite male runner were analyzed over a four-year period (2014–2017). Five of the ten races (50%) were classified as positive split and five (50%) as negative split. Finishing times ranged from 69.18 to 87.05 min. Total ascent ranged from 6.5 to 242.7 m, total descent from 99.7 to 235.1 m, and elevation range from 26.3 to 116.8 m. The absolute between-segment time difference ranged from 0.03 to 2.54 min. When expressed relative to total race time, proportional differences ranged from 0.04% to 2.92% (**Table 1**). Race-specific pacing profiles and elevation characteristics are presented in **Table 1**, while stratified comparisons by pacing classification are shown in **Table 2**. Of the four fastest performances (<73 min), two were classified as positive split and two as negative split. Race 7 exhibited the highest total ascent (242.7 m) and was classified as positive split, with a proportional segment difference of 0.30%. The fastest performance (69.18 min) occurred during a negative split, whereas the slowest performance (87.05 min) occurred during a positive split. The magnitude and direction of split differences across races are illustrated in **Figure 1**. The relationship between elevation range and finishing time across races is presented in **Figure 2**.

**Table 1:** Race characteristics, elevation metrics, and pacing profiles across ten half-marathon performances.

Race	Finishing time (min)	0–10 km (min)	11–21 km (min)	Segment difference* (min)	Segment difference (%)	Total ascent (m)	Total descent (m)	Elevation range (m)	Pacing profile
1	87.05	40.03	42.57	2.54	2.92	152.2	150.7	85.5	Positive
2	84.43	39.24	40.31	1.07	1.27	107.2	108.7	62.6	Positive
3	72.21	34.19	34.42	0.23	0.32	126.1	124.1	30.5	Positive
4	71.59	33.46	34.16	0.7	0.98	6.5	114.8	116.8	Positive
5	72.5	34.47	34.21	-0.26	0.36	97.4	99.7	63.9	Negative
6	69.18	33.5	32.48	-1.02	1.47	123.9	127.2	30.8	Negative
7	76.51	36.1	36.33	0.23	0.30	242.7	235.1	82.3	Positive
8	73.28	35.36	34.43	-0.93	1.27	135.6	134.6	84.0	Negative
9	72.29	34.3	34.27	-0.03	0.04	99.9	102.8	63.0	Negative
10	73.35	34.51	34.15	-0.36	0.49	152.1	154.1	26.3	Negative

*Segment difference calculated as (11–21 km split minus 0–10 km split). Positive values indicate slower second-half performance; negative values indicate faster second-half performance.

**Table 2:** Comparison of elevation and performance variables by pacing strategy.

Variable	Positive split (n = 5)	Negative split (n = 5)
Finish Time (min)	78.3 (72.2 – 87.1)	72.7 (69.2 – 76.5)
Elevation Range (m)	84.5 (30.5 – 116.8)	52.3 (26.3 – 84.0)
Total Ascent (m)	147.0 (107.2 – 242.7)	117.4 (97.4 – 152.1)
Segment Difference (min)	+0.48 (+0.23 to +2.54)	-0.26 (-1.02 to -0.03)

Values are presented as median (range). No inferential statistical testing was performed.

## 4 Discussion

This longitudinal case report examined how moderate elevation variability was associated with pacing behavior and performance outcomes in an elite half-marathon runner. Across ten official road races, pacing strategies were evenly distributed between positive and negative split classifications. Performance outcomes were not uniformly aligned with a single pacing profile: although the fastest performance (69.18 min) occurred during a negative split on a near-flat course, two of the four fastest races were classified as positive split. These findings indicate that pacing direction alone did not consistently determine performance outcomes within this athlete.

The relationship between elevation characteristics and pacing behavior was similarly non-uniform. Within elevation ranges of approximately 60–80 m, both positive and negative pacing profiles were observed, and negative split races within this range were not uniformly slower or faster than positive split races. Finishing times varied substantially despite similar elevation variability. The race with the highest elevation range was among the faster performances, whereas other races with similar variability were slower. Moreover, Race 7, which exhibited the greatest total ascent, demonstrated only a 0.30% proportional segment difference and a mid-range finishing time. These contrasts suggest that elevation metrics alone, whether range or total ascent, do not fully account for pacing classification or performance differences in this dataset. Instead, terrain characteristics likely interacted with contextual, physiological, and race-specific factors.

Nevertheless, the potential influence of terrain on pacing regulation remains physiologically plausible. Uphill running increases metabolic cost and cardiovascular strain, whereas downhill running involves eccentric muscle loading that may impair neuromuscular efficiency and elevate perceived exertion during later race stages (Minetti et al., 2002; Vernillo et al., 2017; Bontemps et al., 2020). Such biomechanical and physiological responses are well documented in trail and mountain running (Saugy et al., 2013; Jaén-Carrillo et al., 2025), where terrain strongly shapes pacing strategies. In urban road races with mild undulations, these mechanisms may operate in a subtler and more context-dependent manner. Previous group-level investigations have reported that negative or even pacing is frequently associated with superior endurance performance (Foster et al., 1994; Abbiss and Laursen, 2008; Tucker and Noakes, 2009; Hanley, 2016). More recent work has highlighted the importance of pacing stability and reduced variability for competitive success across endurance events (Oficial-Casado et al., 2022; Fariod et al., 2023; Cuk et al., 2024; Sha et al., 2024). However, these associations are typically derived from cross-sectional or large-cohort analyses. The present within-athlete longitudinal observations indicate that such relationships may not manifest uniformly across competitive contexts, underscoring the individualized and multifactorial nature of pacing regulation.

From a psychophysiological perspective, negative split pacing has been linked to improved thermoregulatory efficiency, favorable perception of effort, and enhanced tactical execution (Casado et al., 2021; Girardi et al., 2022). In the present dataset, some races exhibited very small proportional between-segment differences, likely reflecting near-even pacing within the two-segment analytical framework. When the slowest race is excluded, all remaining performances show pacing differences below approximately 1.5% between segments, with most below 1%. This pattern suggests that the athlete was generally able to maintain a near-even pacing strategy across races despite differences in course elevation characteristics. Such stability in pacing is consistent with previous observations indicating that elite endurance athletes are capable of sustaining highly controlled effort distribution during competition (Abbiss and Laursen, 2008; Hanley, 2016). This supports the view that elite pacing regulation operates along a continuum rather than within strictly binary categories. An important strength of this study lies in its longitudinal, within-athlete design. By analyzing multiple performances from the same elite runner over several years, inter-individual variability related to physiology, training background, and competitive experience was minimized. Such case-based approaches complement large-scale cohort analyses by providing detailed insight into real-world pacing regulation in elite competition (Oficial-Casado et al., 2022; Fariod et al., 2023; Cuk et al., 2024). Given the single-athlete longitudinal design, the present findings should be interpreted as descriptive and hypothesis-generating rather than generalizable, highlighting the need for future studies in larger elite cohorts to confirm these observations.

## 5 Conclusion

This longitudinal case report explored how moderate elevation variability was associated with pacing behavior and performance outcomes in an elite half-marathon runner. Across ten races, pacing classifications and elevation characteristics were not uniformly aligned with finishing performance. Both positive and negative split profiles were observed across comparable elevation ranges. These findings suggest that terrain features may interact with pacing regulation in a context-dependent manner rather than acting as isolated determinants of performance. The results highlight the complexity of pacing behavior in elite competition and support the value of individualized longitudinal analyses. Future research involving larger elite cohorts and integrated physiological monitoring is warranted to further clarify how course profile interacts with pacing execution.

## 6 Limitations

This study presents several limitations that should be acknowledged. First, as a single-subject longitudinal case report, the observations cannot be generalized to broader populations. Although repeated race performances from one elite athlete provide valuable intra-individual insight, they do not capture the variability in pacing responses that may exist across different runners or performance levels. Second, while elevation characteristics were documented for each race, other environmental factors such as ambient temperature, wind conditions, and humidity were not systematically recorded and may have influenced pacing behavior and performance outcomes. Third, no direct physiological measurements (e.g., heart rate, blood lactate concentration) were collected during competition, limiting the ability to directly assess internal load, fatigue development, or thermoregulatory strain during races. Fourth, pacing classification was based on official split times and GPS-derived data, which may be subject to minor measurement inaccuracies. Finally, the athlete’s physical condition and readiness may not have been identical across all ten races. Nevertheless, the relatively consistent competition level, training background, and performance standard across the observation period suggest a stable physiological framework within which pacing adaptations were examined. Additionally, although elevation metrics and split times were analyzed, the within-race distribution of terrain changes (e.g., whether ascent or descent occurred predominantly in the first or second half of the race) was not systematically modeled. Similarly, pacing was examined using a two-segment framework, which does not capture finer pacing dynamics such as fast starts or end spurts. More granular GPS-based segmentation and terrain mapping may provide deeper insight into how course profile interacts with pacing regulation in elite competition. Accordingly, the findings should be interpreted as illustrative and exploratory rather than definitive.

## Data Availability

The datasets analyzed in this study are derived from publicly available official race results. Summary data and derived analyses supporting the findings are included in the article. Further inquiries can be directed to the corresponding author.
